# Dendrimer-based posaconazole nanoplatform for antifungal therapy

**DOI:** 10.1080/10717544.2021.1986605

**Published:** 2021-10-07

**Authors:** Shengzhuang Tang, Jesse Chen, Jayme Cannon, Zhengyi Cao, James R. Baker, Su He Wang

**Affiliations:** Department of Internal Medicine, Division of Allergy, Michigan Nanotechnology Institute for Medicine and Biological Sciences, University of Michigan, Ann Arbor, MI, USA

**Keywords:** Demethylase inhibitor, antifungal drug, biocompatible, polymer

## Abstract

We examined formulating a new antifungal agent, posaconazole (POS) and its derivatives, with different molecular vehicles. Several combinations of drug and carrier molecules were synthesized, and their antifungal activities were evaluated against *Aspergillus fumigatus*. Posaconazole and four of its derivatives were conjugated to either generation 5 (G5) dendrimers or partially modified G5 dendrimers. The *in vitro* antifungal activities of these compounds suggest that conjugates with specific chemical linkages showed better fungistatic activity than direct conjugates to POS. In particular, a polyethylene glycol (PEG)-imidazole modified G5 dendrimer demonstrated improved antifungal efficacy relative to the parent G5 molecule. Further studies were then conducted with POS derived molecules coupled to PEG-imidazole modified G5 dendrimers to achieve a highly soluble and active conjugate of POS. This conjugated macromolecule averaged 23 POS molecules per G5 and had a high solubility with 50 mg/mL, which improved the molar solubility of POS from less than 0.03 mg/mL to as high as 16 mg/mL in water. The primary release profile of the drug in human plasma was extended to over 72 h, which is reflected in the *in vitro* inhibition of *A. fumigatus* growth of over 96 h. These POS–polymer conjugates appear to be novel and efficient antifungal agents.

## 1. Introduction

Fungal infections are responsible for millions of human deaths each year (Ikeh et al., 2017). Developing new antifungal agents and improving the existing ones remains an important worldwide goal (Filipczak et al., 2021). Current antifungal agents, such as amphotericin B (AmB), echinocandins, flucytosine, and triazole antifungals, all have low aqueous solubility. These agents require long-term and high dose treatment, leading to a limited spectrum of activity and problems with toxicity and pharmacokinetics (Campoy & Adrio, 2017).

Posaconazole (POS) is a broad-spectrum, second generation, triazole compound. It is a lanosterol 14α-demethylase inhibitor (Munayyer et al., 2004; Nagappan & Deresinski, 2007) that has been utilized for the treatment and prophylaxis of invasive *Aspergillus* and *Candida* infections in immunocompromised patients. It is particularly important for treatment of fungal infections found to be refractory to other antifungal therapy. As with other antifungal hydrophobic structures though, POS has poor solubility in water, limited to approximately 0.027 mg/L at 25 °C (United States Enivronmental Protection Agency, 2004). Posaconazole oral dosing is recommended to be given with foods or a nutritional supplement to enhance uptake; despite this, there are still issues with inconsistent absorption, metabolism, elimination, and drug–drug interactions of this compound (Greer, 2007).

To address these issues, POS has been formulated into an oral suspension, a delayed-release tablet, or an intravenous form (European Medicines Agency, 2014). However, the quality of the formulations can vary greatly based on the approach, and stability problems have been observed as well. Additionally, oral formulations have exhibited significant variations in bioavailability in different patients (Tang, 2017). Furthermore, achieving high doses is difficult given the drug’s hydrophobic nature and unintended accumulation of POS in various tissues could lead to unexpected toxicity. To improve POS bioavailability, development of a new solubilizing formulation is of particularly importance.

Polyamidoamine (PAMAM) dendrimers have been extensively utilized in drug formulations due to their well-defined structure, high solubility, and the possession of many reactive surface functional groups (Tomalia et al., 1990; Lee et al., 2005; Soliman et al., 2011), all of which have been found to improve drug delivery (Esfand & Tomalia, 2001). Our previous works have utilized the PAMAM generation 5 (G5) dendrimer platform, maximizing G5’s increase in conjugation sites while avoiding filtration issues due to particle size, to create targeted breast cancer image-enhancing nanocompounds for both CT and MR imaging (Otis et al., 2016; Chen et al., 2020). Furthermore, we also have successfully designed a nanoscale reactor, by the conjugation of partially modified polyethylene glycol (PEG)-imidazole G5 dendrimer with an organophosphate (OP)-reactive α-nucleophile terminal, to inactivate paraoxon-ethyl (POX) for treatment of reactive OP intoxication (Wong et al., 2020).

Here, we propose to conjugate POS to G5 dendrimers to enhance its bioavailability. We initially developed four POS derivatives and investigated their efficacy against *Aspergillus fumigatus*. Next, to improve antifungal activity, both POS and POS derivatives were coupled to G5 dendrimer. Finally, to find the most potent formulation, multiple modifications of G5 dendrimer and POS-conjugation strategies were synthesized and evaluated to identify the lead G5 conjugate (**II-5**).

## 2. Materials and methods

### 2.1. Materials

All solvents and chemicals were purchased from Sigma-Aldrich (St. Louis, MO) and used as received. Phosphate-buffered saline without calcium and magnesium was from Thermo Scientific (Logan, UT). 10,000 MWCO dialysis membrane was from Spectrum Laboratories (Rancho Dominguez, CA). Posaconazole (Noxafil, Schering Corporation, Kenilworth, NJ) was purchased from Biosynth Carbosynth^®^ (catalog no. FP27107). The G5 PAMAM dendrimer was from Dendritech (Midland, MI) and purified by dialysis (10,000 MWCO dialysis membrane) against H_2_O. The number of terminal amino groups on G5 dendrimer is experimentally 114 (Choi et al., 2012; Mukherjee et al., 2015).

### 2.2. Preparation of G5-PEGim

G5 dendrimer was partially modified with PEG-imidazole via carbonyl diimidazole (CDI) coupling chemistry, as done in our previous work (Wong et al., 2020); additional methods are also in Supporting Information-2.

### 2.3. Synthesis of posaconazole derivatives

#### 2.3.1. Activation of posaconazole with 4-nitrophenyl chloroformate

To a solution of POS (0.245 g, 0.35 mmol) dissolved in dry dichloromethane (20 mL) were added 4-(dimethylamino)pyridine (DMAP) (0.513 g, 4.2 mmol) and 4-nitrophenyl chloroformate (0.846 g, 4.2 mmol). The mixture was stirred at room for overnight. White precipitate was filtered off and washed with DCM (10 mL). The filtrates were combined, washed with sat. NaHCO_3_ (20 mL), 10% KHSO_4_ (20 mL), and brine (20 mL), and then dried over Na_2_SO_4_. After concentration, the residue was purified by flash column chromatography by eluting with 5% methanol in dichloromethane. The compound **I**, POS nitrophenyl carbonate was obtained as an off-white fluffy solid (0.278 g, yield 92%). *Rf* (5:100 methanol/dichloromethane)=0.38. ESI-MS *m/z*: 866.3422 [M + H]^+^ C_44_H_45_F_2_N_9_O_8_ (865.3359). ^1^H NMR (500 MHz, CDCl_3_): *δ* 8.06–8.05 (d, *J* = 5.0 Hz, 2H (nitrophenyl)), 7.98 (s, 1H), 7.71 (s, 1H), 7.50 (s, 1H), 7.27–7.18 (m, 3H), 6.93–6.92 (d, *J* = 5.0 Hz, 2H (nitrophenyl)), 6.95–6.79 (m, 3H), 6.73–6.64 (m, 3H), 4.99–4.96 (m, 1H, (pos)CH–O(C=O)), 4.08–4.04 (m, 1H), 3.94–3.90 (m, 2H), 3.66–3.61 (m, 2H), 3.56–3.53 (m, 2H), 3.20–3.18 (m, 4H), 3.05–3.03 (m, 4H), 2.42–2.32 (m, 2H), 2.20–1.94 (m, 1H), 1.82–1.75 (m, 1H), 1.69–1.66 (m, 1H), 1.28–1.27 (d, *J* = 5.0 Hz, 3H)), 0.74–0.71 (t, *J* = 7.5 Hz, 3H) ppm.

#### 2.3.2. Synthesis of derivative A–D

Active compound **I** (1 equivalent) was dissolved in minimum methanol and added to cooled aqueous pH 10 solution containing N-terminal small molecules (2 equivalent) (100 mg/mL) (glycine for **A**; serine for **B**; ethanolamine for **C**; 2-(2-aminoethoxy)ethanol for **D**). After TLC showed **I** was consumed completely, the reaction mixture was adjusted to pH 7 with 0.2 M HCl and then extracted twice with DCM (equal volume with reaction solution). The DCM layers were combined, dried over Na_2_SO_4_, then concentrated and purified by flash column chromatography using methanol in DCM as eluting solution.

*Derivative A:* Concentrated and purified on a column eluting with 10% methanol in dichloromethane. Yield 59%. *Rf* (20% methanol in dichloromethane)=0.45. HPLC analysis: RT = 8.975 min (purity ≥95%). ESI-MS *m/z*: 802.3487 [M + H]^+^; 800.3320 [M–H]^–^ C_40_H_45_F_2_N_9_O_7_ (801.3410). ^1^H NMR (500 MHz, CDCl_3_): *δ* 8.14 (s, 1H), 7.81 (s, 1H), 7.71 (s, 1H), 7.41–7.37 (m, 3H), 7.00–6.98 (d, *J* = 10.0 Hz, 2H), 6.98 (br, 2H), 6.88–6.82 (m, 2H), 6.79–6.77 (d, *J* = 10.0 Hz, 2H), 5.94 (br s, 1H, –NH–(C=O)–O–), 5.06–5.03 (m, 1H, (pos)CH–O(C=O)), 4.66–4.63 (d, *J* = 15.0 Hz, 1H), 4.52–4.49 (d, *J* = 15.0 Hz, 1H), 4.18 (br, 1H), 4.12–4.10 (t, *J* = 5.0 Hz, 1H), 3.94–3.84 (m, 2H (glycine)), 3.79–3.76 (t, *J* = 6.5 Hz, 1H), 3.71–3.68 (t, *J* = 6.5 Hz, 1H), 3.62–3.59 (t, *J* = 6.5 Hz, 1H), 3.71–3.69 (m, 1H), 3.37 (br s, 4H), 3.25 (br s, 4H), 2.64–2.53 (m, 2H), 2.10–2.06 (m, 1H), 1.93–1.87 (m, 1H), 1.80–1.78 (m, 1H), 1.33–1.32 (d, *J* = 5.0 Hz, 3H), 0.91–0.88 (t, *J* = 7.5 Hz, 3H) ppm. ^13^C (500 MHz, CDCl_3_): 171.84, 160.05, 155.74, 153.79,150.90, 144.56, 135.26, 128.61, 125.19, 124.23, 118.71, 116.51, 115.21, 111.43, 111.29, 104.87, 104.66, 104.46, 84.02, 71.94, 70.76, 68.97, 60.73, 55.98, 50.78, 48.92, 42.69, 38.86, 37.42, 22.60, 17.91, and 10.42 ppm.

*Derivative B:* Concentration and purified on a column eluting with 20% methanol/dichloromethane. Yield 62%. *Rf* (30% methanol in dichloromethane)=0.30. HPLC analysis: RT = 8.816 min (purity ≥95%). ESI-MS *m/z*: 832.3585 [M + H]^+^; 854.3392 [M + Na]^+^ C_42_H_51_F_2_N_9_O_7_ (831.3516). ^1^H NMR (500 MHz, CD_3_OD): 8.38 (s, 1H), 8.09 (s, 1H), 7.77 (s, 1H), 7.46–7.44 (d, *J* = 10.0 Hz, 2H), 7.41–7.36 (m, 1H), 7.13–7.11 (d, *J* = 10.0 Hz, 2H), 7.01–6.98 (m, 3H), 6.89–6.86 (t, *J* = 7.5 Hz, 2H), 6.83–6.81 (d, *J* = 10.0 Hz, 2H), 5.06–5.03 (m, 1H, (pos)CH–O(C=O)), 4.67 (s, 2H), 4.14–4.11 (t, *J* = 7.5 Hz, 2H), 3.86–3.83 (t, *J* = 7.5 Hz, 1H), 3.79–3.76 (t, *J* = 7.5 Hz, 1H), 3.73–3.70 (t, *J* = 7.5 Hz, 1H), 3.57–3.17 (m, 9H(POS) and 3H(serine)), 2.69–2.52 (m, 2H), 2.22–2.17 (m, 1H), 1.94 (br m, 1H), 1.83 (br m, 1H), 1.30–1.29 (d, *J* = 5.0 Hz, 3H), 0.89–0.86 (t, *J* = 7.5 Hz, 3H) ppm. ^13^C NMR (500 MHz, CD_3_OD): *δ* 154.91, 152.41, 147.08, 137.29, 129.92, 126.65, 125.32, 119.85, 117.52, 116.23, 112.15, 111.95, 105.37, 105.15, 85.18, 73.09, 71.57, 70.11, 62.36, 57.15, 52.10, 50.14, 40.33, 38.91, 23.24, 17.95, and 10.78 ppm.

*Derivative C:* Concentrated and purified on a column eluting with 5% methanol in dichloromethane. Yield 88%. *Rf* (5:100 methanol/dichloromethane)=0.40. HPLC analysis: RT = 8.933 min (purity ≥95%). ESI-MS *m/z*: 788.3678 [M + H]^+^ C_40_H_47_F_2_N_9_O_6_ (787.3617). ^1^H NMR (500 MHz, CDCl_3_): 8.11 (s, 1H), 7.80 (s, 1H), 7.63 (s, 1H), 7.43–7.36 (m, 3H), 7.03–7.01 (d, *J* = 10.0 Hz, 2H), 6.93 (br s, 2H), 6.87–6.77 (m, 4H), 5.35 (br s, 1H, –NH–(C=O)–O–), 5.10–5.08 (m, 1H, (pos)CH–O(C=O)), 4.66–4.63 (d, *J* = 15.0 Hz, 1H), 4.52–4.49 (d, *J* = 15.0 Hz, 1H), 4.16–4.10 (m, 2H), 3.79–3.76 (t, *J* = 5.0 Hz, 1H), 3.71–3.69 (m, 1H), 3.62–3.59 (m, 3H, 1H-POS and CH_2_–N (ethanolamine), 3.37 (br s, 4H), 3.25 (br s, 6H, 4H-POS and CH_2_–O (ethanolamine)), 2.64–2.53 (m, 2H), 2.10–2.06 (m, 1H), 1.98–1.92 (m, 1H), 1.80–1.79 (m, 1H), 1.32–1.31 (d, *J* = 5.0 Hz, 3H), 0.91–0.88 (t, *J* = 7.5 Hz, 3H) ppm. ^13^C NMR (500 MHz, CDCl_3_): 156.62, 153.29, 151.13, 144.57, 134.43, 128.61, 123.49, 118.48, 116.63, 115.20, 111.42, 111.26, 104.84, 104.64, 104.43, 84.08, 84.05, 72.05, 70.75, 68.95, 62.14, 60.91, 55.95, 50.64, 49.09, 43.39, 38.84, 37.43, 22.26, 17.74, and 10.51 ppm.

*Derivative D:* Concentrated and purified on a column eluting with 5% methanol in dichloromethane. Yield 86%. *Rf* (10% methanol/dichloromethane)=0.15. HPLC analysis: RT = 8.991 min (purity ≥95%). ESI-MS *m/z*: 832.4014 [M + H]^+^; 854.3756 [M + Na]^+^ C_42_H_51_F_2_N_9_O_7_ (831.3880). ^1^H NMR (500 MHz, CDCl_3_): 8.11 (s, 1H), 7.80 (s, 1H), 7.65 (s, 1H), 7.44–7.36 (m, 3H), 7.04–7.02 (d, *J* = 10.0 Hz, 2H), 6.93 (br s, 2H), 6.87–6.77 (m, 4H), 5.38 (br s, 1H, –NH–(C=O)–O–), 5.11–5.08 (m, 1H, (pos)CH–O(C=O)), 4.66–4.63 (d, *J* = 15.0 Hz, 1H), 4.52–4.49 (d, *J* = 15.0 Hz, 1H), 4.16–4.09 (m, 2H), 3.79–3.76 (t, *J* = 5.0 Hz, 1H), 3.71–3.68 (m, 1H), 3.62–3.59 (m, 3H, 1H-POS and CH_2_–N (aminoethoxyethanol)), 3.50–3.46 (br m, 4H, –CH_2_–O–CH_2_–(aminoethoxyethanol)), 3.36 (br s, 4H), 3.31–3.30 (br m, 2H, –CH_2_–O(terminal aminoethoxyethanol)), 3.23 (br s, 4H), 2.64–2.49 (m, 2H), 2.10–2.04 (m, 1H), 1.97–1.93 (m, 1H), 1.80–1.61 (m, 1H), 1.30–1.29 (d, *J* = 5.0 Hz, 3H), 0.91–0.88 (t, *J* = 7.5 Hz, 3H) ppm. ^13^C NMR (500 MHz, CDOD_3_): 156.01, 153.29, 151.14, 144.61, 134.38, 128.67, 128.62, 128.55, 123.53, 118.61, 116.75, 115.24, 111.44, 111.26, 104.86, 104.66, 104.45, 84.10, 84.07, 72.33, 71.77, 70.76, 69.98, 68.97, 61.62, 60.88, 55.93, 50.91, 49.02, 40.81, 38.85, 37.43, 22.28, 17.77, and 10.52 ppm.

#### 2.3.3. Activation of posaconazole derivative D with 4-nitrophenyl chloroformate

To a solution of derivative **D** (0.135 g, 0.16 mmol) dissolved in dry dichloromethane (5 mL) were added 4-(dimethylamino)pyridine (0.117 g, 0.96 mmol) and 4-nitrophenyl chloroformate (0.193 g, 0.96 mmol). The mixture was stirred overnight at room temperature. White precipitate was filtered off and washed with DCM (5 mL). The filtrates were combined, washed with sat. NaHCO_3_ (5 mL), 10% KHSO_4_ (5 mL), and brine (5 mL), and then dried over Na_2_SO_4_. After concentration, the residue was purified by flash column chromatography by eluting with 5% methanol in 1:1 dichloromethane/ethylacetate. The compound **II**, N-(POS-carbonyl)diglycolamine-nitrophenyl carbonate was obtained as a white fluffy solid (0.278 g, yield 88**%)**. *Rf* (5% methanol in dichloromethane/ethylacetate)=0.30. ESI-MS *m/z*: 997.3987 [M + H]^+^ C_49_H_54_F_2_N_10_O_11_ (996.3942). ^1^H NMR (500 MHz, CD_3_OD): *δ* 8.38 (s, 1H), 8.28–8.26 (d, *J* = 10.0 Hz, 2H (nitrophenyl)), 8.09 (s, 1H), 7.77 (s, 1H), 7.44–7.35 (m, 3H), 7.11–7.09 (d, *J* = 10.0 Hz, 2H (nitrophenyl)), 7.02–6.94 (m, 3H), 6.89–6.80 (m, 3H), 5.03–5.00 (m, 1H, (pos)CH–O(C=O)), 4.67 (m, 1H), 4.34 (m, 1H), 4.14–4.08 (m, 2H), 3.87–3.67 (m, 5H), 3.50–3.44 (m, 2H), 3.35–3.15 (m, 12H), 2.62–2.52 (m, 2H), 2.21–2.17 (m, 1H), 1.93–1.88 (m, 1H), 1.84–1.80 (m, 1H), 1.29–1.28 (d, *J* = 5.0 Hz, 3H), and 0.89–0.86 (t, *J* = 7.5 Hz, 3H) ppm.

### 2.4. Synthesis of G5 dendrimer posaconazole conjugates: general procedure for conjugation and purification

Active compound **I**, POS nitrophenyl carbonate or **II**, N-(POS-carbonyl)diglycolamine-nitrophenyl carbonate was dissolved in a minimal volume of DCM. With the specific molar ratio of POS to dendrimer (non-modified or imidazole-modified) ([activated compound **I** or **II**]/[dendrimer] = 10, 20, 30), the solution was added to the rapidly stirred solution of dendrimer dissolved in methanol (5 mg/mL) at 0 °C in the presence of N,N-diisopropylethylamine ([DiPEA]/[dendrimer] = 300). The reaction mixture was warmed to room temperature and stirred overnight. The reaction mixture was poured into acetonitrile (six times the volume to reaction solution) and cooled to 3–5 °C. After removing the solvents, the residue was diluted with adequate PBS (pH 7.4) (dendrimer/PBS = 5 mg/mL), loaded into cellulose membrane dialysis tubing (MWCO 10 kDa), and dialyzed against deionized water (2×), PBS (2×), deionized water (1×), PBS (1×), and deionized water (4×) over three days. The purified solution in the dialysis bags was filtered through a 0.22 μm filter unit, collected, and lyophilized to afford desired G5-POS conjugates as white fluffy solid.

#### 2.4.1. Class I of G5 dendrimer posaconazole conjugates

**I-1** ((POS)6-G5): compound **I** (8 mg), G5 (25 mg), DiPEA (58 µL), methanol (5 mL). **I-1** yield: 29 mg. MALDI-TOF (*m/z*): 29,731 g mol^−1^. ^1^H NMR (500 MHz, D_2_O): *δ* 8.28 (br s), 8.01 (br s), 7.83 (br s), 7.39 (br s), 7.11–6.74 (br m), 4.94 (br),4.15 (br s), 3.53 (br s), 3.35 (br s), 3.17 (br s), 3.16 (br s), 3.02–2.88 (br m), 2.68 (br s), 2.53 (br s), 2.47 (br s), 1.84 (br s), 1.27 (br s, –CH_3_ (POS)), and 0.79 (br s, –CH_3_ (POS)) ppm.

**I-2** ((POS)5.2-G5-PEGim): compound **I** (4.4 mg), G5-PEGim (16 mg), DiPEA (29 µL), methanol (3.2 mL). **I-2** yield: 17 mg. MALDI-TOF (*m/z*): 32,009 g mol^−1^. ^1^H NMR (500 MHz, D_2_O): *δ* 8.27 (br s), 8.01 (br s), 7.83 (br s), 7.40 (br s), 7.24–6.74 (br m), 4.95 (br), 4.21 (br s, –OCH_2_– (PEGim)), 4.13 (br, s), 3.71 (br s), 3.55 (br s), 3.48 (br s), 3.35 (br s), 3.19 (br s), 3.18 (br s), 2.89 (br s), 2.69 (br s), 2.54–2.27 (br m), 2.03 (br s, –CH_2_– (PEGim)), 1.83 (br s), 1.27 (br s, –CH_3_ (POS)), and 0.77 (br s, –CH_3_ (POS)) ppm.

#### 2.4.2. Class II of G5 dendrimer posaconazole conjugates

**II-1** (G5-(POS)5.6): compound **II** (12.3 mg), G5 (33 mg), DiPEA (65 µL), methanol (6.6 mL). **I-2** yield: 44 mg. MALDI-TOF (*m/z*): 30,075 g mol^−1^. ^1^H NMR (500 MHz, D_2_O): *δ* 8.29 (br s), 8.02 (br s), 7.80 (br s), 7.40 (br s), 7.11–6.76 (br m), 4.95 (br), 4.08 (br, s), 3.71 (br s), 3.53 (br s), 3.52 (br s), 3.34 (br s), 3.16–3.14 (br m), 2.89 (br s), 2.87 (br s), 2.68 (br s), 2.52–2.27 (br m), 1.84 (br s), 1.28 (br s, –CH_3_ (POS)), and 0.78 (br s, –CH_3_ (POS)) ppm.

**II-2** (G5-PEGim-(POS)6.8): compound **II** (18.8 mg), G5-PEGim (54.7 mg), DiPEA (99 µL), methanol (11 mL). **II-2** yield: 62 mg. MALDI-TOF (*m/z*): 34,238 g mol^−1^. ^1^H NMR (500 MHz, D_2_O): *δ* 8.29 (br), 8.03 (br), 7.80 (br), 7.40 (br s), 7.23–6.66 (br m), 4.22 (br s, –OCH_2_– (PEGim)), 4.10 (br, s), 3.72 (br s), 3.54 (br s), 3.35 (br s), 3.17 (br s), 2.88 (br s), 2.68 (br s), 2.53 (br s), 2.47 (br s), 2.02 (br s, –CH_2_– (PEGim), 1.84 (br), 1.27 (br, –CH_3_ (POS)), and 0.77 (br, –CH_3_ (POS)) ppm.

**II-3** (G5-PEGim-(POS)16): compound **II** (33 mg), G5-PEGim (48 mg), DiPEA (88 µL), methanol (9.6 mL). **II-3** yield: 57 mg. 42,413 g mol^−1^. ^1^H NMR (500 MHz, D_2_O): *δ* 8.20 (br), 7.87–7.73 (br m), 7.47 (br), 7.32 (br), 7.16 (br), 6.84 (br), 6.84 (br), 4.30 (br, s), 4.21 (br s), 3.80 (br), 3.61 (br s), 3.42 (br s), 3.24 (br s), 2.96 (br s), 2.76 (br s), 2.61 (br s), 2.55 (br s), 2.11 (br), 1.28 (br, –CH_3_ (POS)), and 0.79 (br, –CH_3_ (POS)) ppm.

**II-4** (G5-PEGim-(POS)23): compound **II** (50 mg), G5-PEGim (48 mg), DiPEA (88 µL), methanol (9.6 mL). **II-4** yield: 72 mg. MALDI-TOF (*m/z*): 47,800 g mol^−1^. ^1^H NMR (500 MHz, D_2_O): *δ* 8.30–8.07 (br m), 7.84–7.78 (br m), 7.46 (br), 7.31 (br), 7.16 (br), 6.75 (br), 4.48 (br, s), 4.29–4.20 (br m), 3.81–3.79 (br m), 3.63 (br s), 3.42 (br s), 3.24 (br s), 2.95 (br s), 2.76 (br s), 2.60 (br s), 2.54 (br s), 2.10 (br), 1.30 (br, –CH_3_ (POS)), and 0.80 (br, –CH_3_ (POS)) ppm.

#### 2.4.3. II-5 (G5-Ac-PEGim-(POS)23)

To **II-4** (25 mg) dissolved in methanol (5 mL) was added N,N-diisopropylethylamine (DiPEA) (25 µL), followed by the addition of acetic anhydride (5.5 mg) in methanol (0.1 mL), while rapidly stirring. The reaction mixture was stirred overnight at room temperature. After removal of the organic solvent, the residue was dissolved in water (5 mL) and purified by dialysis against PBS (2×), deionized water (4×) with a membrane dialysis tubing (MWCO 10 kDa). The desired product was lyophilized and produced 20 mg as white fluffy solid. MALDI-TOF (*m/z*): 49,472 g mol^−1^. ^1^H NMR (500 MHz, D_2_O): *δ* 8.18 (br), 7.74 (br), 7.44 (br), 6.75 (br), 4.36–4.09 (br m), 3.97–3.77 (br m), 3.69 (br s), 3.60 (br s), 3.38 (br s), 2.91 (br s), 2.80 (br s), 2.76 (br s), 2.72 (br s), 2.51 (br s, –CH_3_(Ac)), 2.20 (br), 1.27 (br, –CH_3_ (POS)), and 0.80 (br, –CH_3_ (POS)) ppm.

### 2.5. NMR spectroscopy

A 500 MHz Varian spectrometer tellurium 500 MHz was employed for acquiring ^1^H and ^13^C NMR spectra. Chemical shift values (*δ*) are reported in ppm relative to an internal standard (*δ* = 0.00 ppm) such as tetramethylsilane (TMS) in CDCl_3_ or 2,2-dimethyl-2-silapentane-5-sulfonate-d6 (DSS) in D_2_O.

### 2.6. HPLC

HPLC was employed for analyzing the derivatives and conjugates. It was performed on a Waters Acquity System using a photodiode array detector (Milford, MA) (detection at 265 nm). Each sample solution (3 µL, 0.2–0.5 mg/mL) was injected into a BEH300 C4 column (100 × 2.1 mm, 1.7 µm), and elution was performed at a flow rate of 0.2 mL/min with a linear gradient mode using two mobile solvents, eluent A (0.1% TFA/water (v/v)) and B (0.1% TFA/acetonitrile (v/v)). The sample elution began with a mobile phase 1% B (0–2.0 min) which was followed by a linear increase to 80% B (13.4 min), a decrease to 50% B (13.8 min), a decrease to 1% B (14.4 min) and finally an isocratic elution at 1% B (18 min).

### 2.7. Mass spectrometry

Mass analysis of dendrimer conjugates was conducted by matrix-assisted laser desorption ionization time-of-flight mass spectrometry (MALDI-TOF MS) in a Tofspec-2E spectrometer (Waters, Milford, MA) running in linear mode with the high mass PAD detector and 2,5-dihydroxybenzoic acid (DHB) in acetone/water (50:50, v/v) was used as the matrix. Exact molecular mass analysis of small molecules was conducted at a high-resolution VG 70-250-S mass.

### 2.8. UV–vis spectroscopy

UV–vis absorption spectra were recorded on a Perkin Elmer Lambda 25 spectrometer (Waltham, MA). Posaconazole standard solutions were accurately prepared with 1:1 methanol/water (1:1) and the calibration curve for determining the number of attached POS was made based on the standard solution’s absorbance at 257 nm. Conjugate solutions for absorbance were also prepared with 1:1 methanol/water (1:1) at 0.1 mg/mL, equivalent to 10–30 µg/mL of POS.

### 2.9. *In vitro* posaconazole release

Posaconazole release profiles in human plasma were monitored by measuring an increase in fluorescence intensity. Fluorescent spectra were recorded in a Fluoromax-2 fluorimeter (Horiba Scientific, Piscataway, NJ). To 2.4 mg of conjugate **II-5** dissolved in 0.5 mL of phosphate buffer (pH 7.4) was added 0.5 mL of plasma from human. The mixture was immediately transferred to a dialysis bag (MWCO = 10 kDa). It was immersed in 19 mL of phosphate buffer (pH 7.4) in a shaking water bath at 37 °C. At predetermined time points, *t* = 0, 0.5, 1, 2, 6, 12, 24, 48, and 72 h, 1.5 mL of the external buffer was withdrawn and replenished with an equal volume of fresh medium. The amount of released POS was analyzed with fluorescence spectrophotometer with the excitation at 260 nm and detection using emission scan (320–480 nm). Conjugate **II-5** in PBS and plasma in PBS were conducted under the same conditions, for comparison.

### 2.10. *In vitro* antifungal activity

Primary proliferation testing on fungi was conducted using *Aspergillus fumigatus*. To prepare *A. fumigatus* stock (SRRC 2006; ATCC), cultures were grown on potato dextrose agar (PDA; per liter: 4 g potato starch, 20 g dextrose, 15 g agar) plates and incubated at 33 °C for three days. Spores were collected, suspended in sterile PBS, and stored at 4 °C until use. Posaconazole and the candidates were suspended in a 1 mg/mL stock solution in sterile water, respectively. While POS, POS derivative **A**–**D**, and class **I** conjugate **1,2** present as suspensions in water, class **II** conjugate **1**, **2**, **3**, **4**, and **5** were clear solutions. The stock solution was diluted with PDA to equivalent to a concentration of 25 µg/mL POS and then immediately plated on 24-well plates. *A. fumigatus* was inoculated (4 × 10^5^ CFU/well) and plates were incubated at 33 °C. Growth was observed at days 1, 2, 3, 4, and 7 after inoculation.

## 3. Results and discussion

### 3.1. Synthesis of posaconazole derivatives

**Scheme 1.**
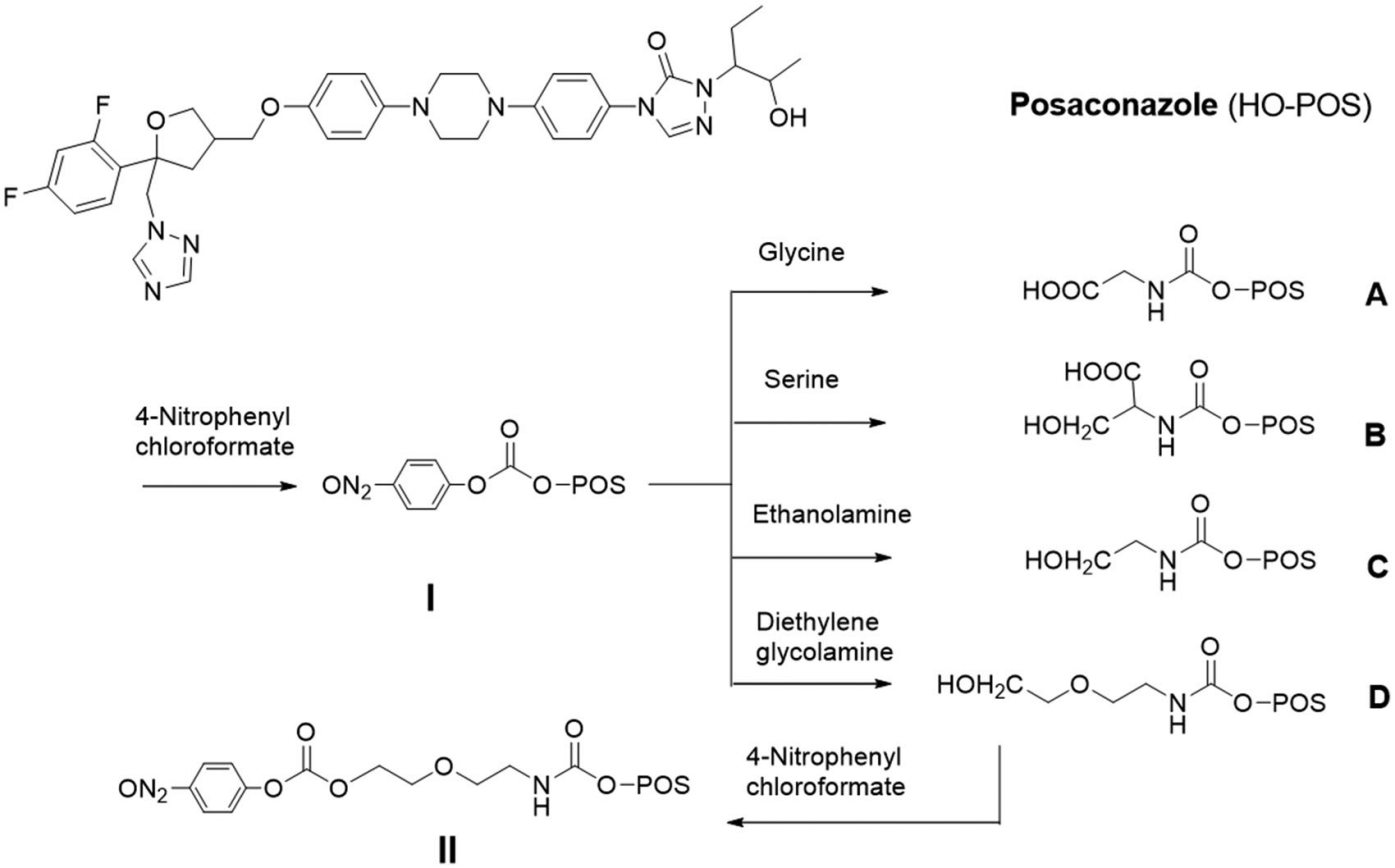
Preparation of POS derivatives **A**–**D**. Conditions: (1) activation with 4-nitrophenyl chloroformate: DMAP,DCM, 16 h; (2) synthesis reaction of derivatives: methanol/water, pH 10.

The active hydroxyl group of POS provides an ideal site for attaching the drug to a G5 dendrimer either directly or through a functional linker. In our initial studies, we conventionally coupled POS directly to G5 dendrimer. However, the outputs showed disappointing antifungal activity. Therefore, we considered attachment of POS through specific linkers to study possible improved fungal inhibition. We designed POS derivatives **A**–**D** by coupling different functional and biocompatible ligands through the hydroxyl group (Scheme 1). POS was activated with 4-nitrophenyl chloroformate in the presence of 4-dimethylaminopyridine (DMAP) to obtain compound **I**, POS nitrophenyl carbonate, which was purified by flash column chromatography. Purified POS nitrophenyl carbonate was reacted with amino-terminal functional ligands to form POS derivatives, or to be directly coupled to surface primary amino groups on a G5 dendrimer. Glycine, l-serine, ethanolamine, and diglycolamine were used as ligands to synthesize derivative **A**, N-(POS-carbonyl)glycine; **B**, N-(POS-carbonyl)l-serine; **C**, N-(POS-carbonyl)ethanolamine; and **D**, N-(POS-carbonyl)diglycolamine; respectively. Herein, N-(POS-carbonyl)glycine retains a bare active carboxyl group; N-(POS-carbonyl)ethanolamine and N-(POS-carbonyl)diglycolamine each retain a bare active hydroxyl group, while N-(POS-carbonyl)l-serine retains both an active carboxyl group and hydroxyl group. This led to the potential to couple these POS derivatives to the G5 dendrimer through these active points. Ultimately, derivative **D**, N-(POS-carbonyl)diglycolamine was activated with 4-nitrophenyl chloroformate to form active compound **II**, N-(POS-carbonyl)diglycolamine-nitrophenyl carbonate, which can be treated with dendrimer to form the indirect conjugates. Each derivative was fully characterized using standard analytical methods that included ^1^H and ^13^C NMR spectroscopy, and mass spectrometry (electrospray ionization mode). The purity analyzed by high-performance liquid chromatography (HPLC) for each derivate was higher than 95% (Supporting Information-1).

### 3.2. Antifungal activities of POS derivatives

POS derivatives **A**–**D** were screened for their preliminary antifungal activity, over a 48 h period *in vitro*, using *A. fumigatus*. The results indicated that the introduction of certain ligands to POS, through the carbamate bond, can change the antifungal activity of POS. Derivatives **A**, N-(POS-carbonyl)glycine and **B**, N-(POS-carbonyl)l-serine with exposed carboxyl groups, showed no inhibition against *A. fumigatus* at 24 h; meanwhile, derivatives **C**, N-(POS-carbonyl)ethanolamine and **D**, N-(POS-carbonyl)diglycolamine with exposed hydroxyl groups, showed improved antifungal activity. Derivative **D** presented the best antifungal effects out of the four derivatives and was also comparable with native POS activity out to 48 h with complete inhibition of fungal growth. This encouraged us to compare the two by coupling POS or derivative **D** with G5 dendrimer to make two classes of conjugates and continue our investigation into their antifungal effects.

### 3.3. Synthesis of POS conjugates with surface modified G5 dendrimers

Two classes of compounds were developed; those were the modified polymers that were directly conjugated to activated POS (i.e. compound **I**, POS nitrophenyl carbonate) or those conjugated indirectly to activated POS derivative **D** (i.e. compound **II**, N-(POS-carbonyl)diglycolamine-nitrophenyl carbonate). All conjugates were purified by precipitating in acetonitrile, followed by membrane dialysis, against deionized water (2×), phosphate-buffered saline (PBS) solution (3×), and back to deionized water (2×), with a molecular weight cutoff (MWCO) of 10 kDa. PEG-imidazole modified G5 dendrimer (G5-PEGim) is prepared as described in Supporting Information-2. Briefly, tetra(ethylene glycol) was first activated with one equivalent of 4-nitrophenyl chloroformate in the presence of *N*,*N*′-di-isopropyl-*N*-ethylamine (DIPEA). The mono-activated tetra(ethylene glycol) was then treated with 1-(3-aminopropyl)imidazole to gain tetraglycol-carbonylaminopropyl)imidazole (PEGim), which was activated with carbonyl diimidazole (CDI) and subsequently treated with G5 dendrimer. The number of PEGim residues attached to G5 was determined by increasing molecular weight of G5-PEGim to G5 detected by matrix-assisted laser desorption ionization-time of flight (MALDI-TOF) mass spectrometry.

**Figure 1.**
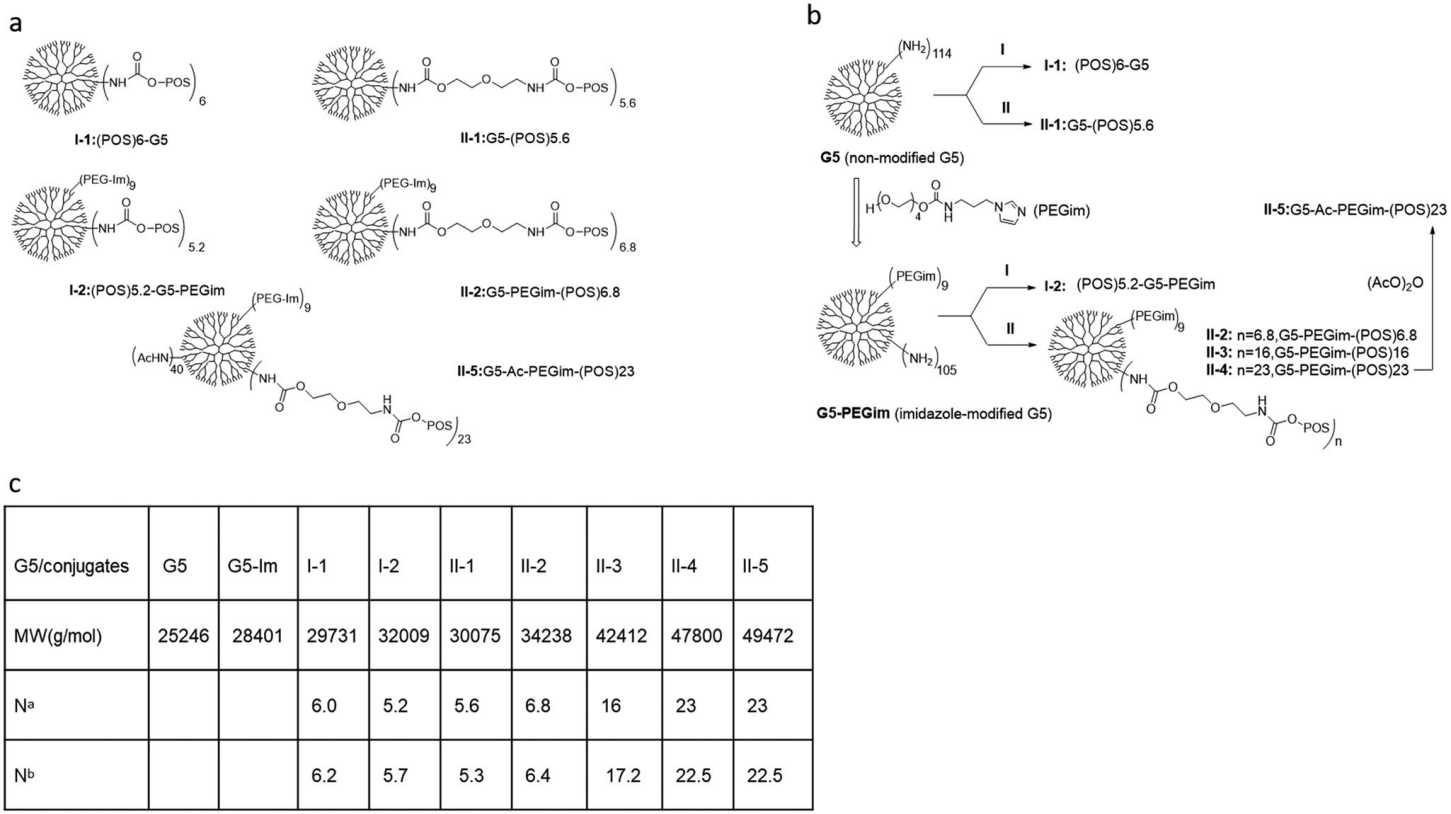
(a) Schematic representation of two classes of conjugates and synthetic targeting conjugate **II-5**. (b) Synthesis of class **I** and **II** of G5 POS conjugates. Conditions: (1) POS coupling reactions and acetylation: DiPEA, MeOH, 0 °C, 2 h; then 16 h, r.t. (2) Imidazole-modification: carbonyl diimidazole (CDI), acetonitrile, 12 h, r.t., and then G5-MeOH, 12 h, r.t. (c) N^a^: POS molecules attached to a G5 dendrimer determined by increasing molecular weights detected by MALDI-TOF spectrometry. N^b^: POS molecules attached to a G5 dendrimer determined by UV–vis spectrometry.

The structures and synthesis protocol of both non-modified G5 or G5-PEGim conjugates is illustrated in Figure 1(a,b). In the class **II** conjugates, increasing the drug load numbers attached on the surface of G5-PEGim was attempted by increasing the ratio of active compound **II** vs. G5 dendrimer. However, at higher loading ratios precipitation occurred during post-synthesis dialysis; a conjugate solution with 23 POS derivative **D** per dendrimer appeared to be the maximum allowable drug per dendrimer ratio, as anything over that led to a cloudy solution.

### 3.4. ^1^H NMR spectroscopy

**Figure 2.**
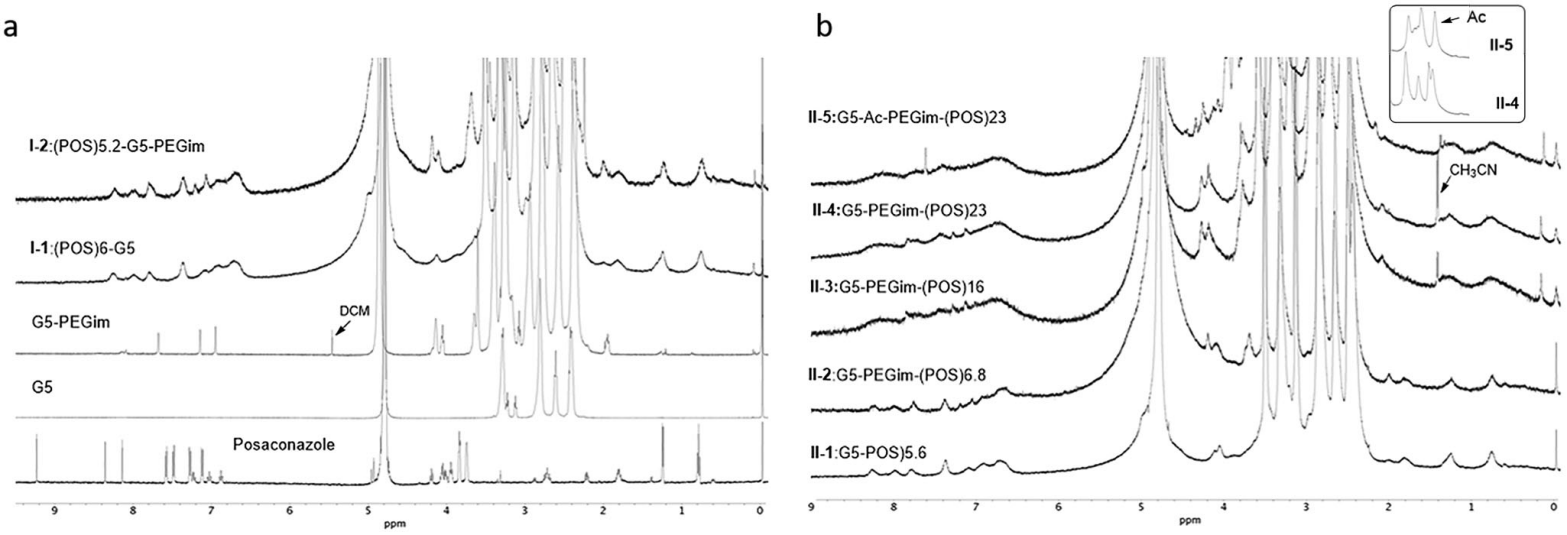
(a) Comparison of ^1^HNMR spectra of POS, non-modified G5, PEG-imidazole modified G5 (G5-PEGim), conjugate **I-1** and **I-2**. (b) Comparison of the ^1^H NMR spectra of conjugate **II-1**, **II-2**, **II-3**, **II-4**, and **II-5**.

^1^H NMR spectrum was used to analyze the synthetic conjugates. As shown in Figure 2(a), compared with G5, G5-PEGim clearly showed three sharp new peaks at 8.0–7.82 ppm, a new set of peaks around 4.0 ppm, and a relatively board peak at about 2.0 ppm, which were all attributed to the attached PEG-imidazole. After direct attachment of POS to dendrimers, both conjugates **I-1** and **I-2** showed POS’s characteristic peaks in the aromatic region and two peaks at approximately 1.2 and 0.8 ppm, which correspond to two methyl groups in POS. Furthermore, as compared to the **I-1** using non-modified **G5** for conjugation, the **I-2** displayed the PEG-imidazole’s unique peaks at both 4 ppm and 2 ppm.

The overlap of the POS’s characteristic peaks in ^1^H NMR spectrum of conjugate **II-1** to **II-5** became increasingly prominent after more molecules were added as shown in Figure 2(b). These peaks were so broad that the numbers of the attached POS could not be determined by comparison of the integration at typical areas. Fortunately, the PEG-imidazole peak at 2 ppm was evident in conjugates **II-2** to **II-5** but absent in conjugate **II-1** since the G5 had no PEG-imidazole.

The ^1^H NMR spectra for the non-acetylated G5 platforms in **II-1** to **II-4** were similar with significant overlap. However, after acetylation in **II-5**, the characteristic peaks for dendrimer changed significantly. A sharp and broad peak at 2.5 ppm was now present indicating that the acetyl groups had covered the dendrimer backbone **(II-5**, the small box above Figure 2(b)). Despite this, the number of acetyl groups could not be determined by NMR due to peak broadening and multiple overlaps.

### 3.5. High-performance liquid chromatography

**Figure 3.**
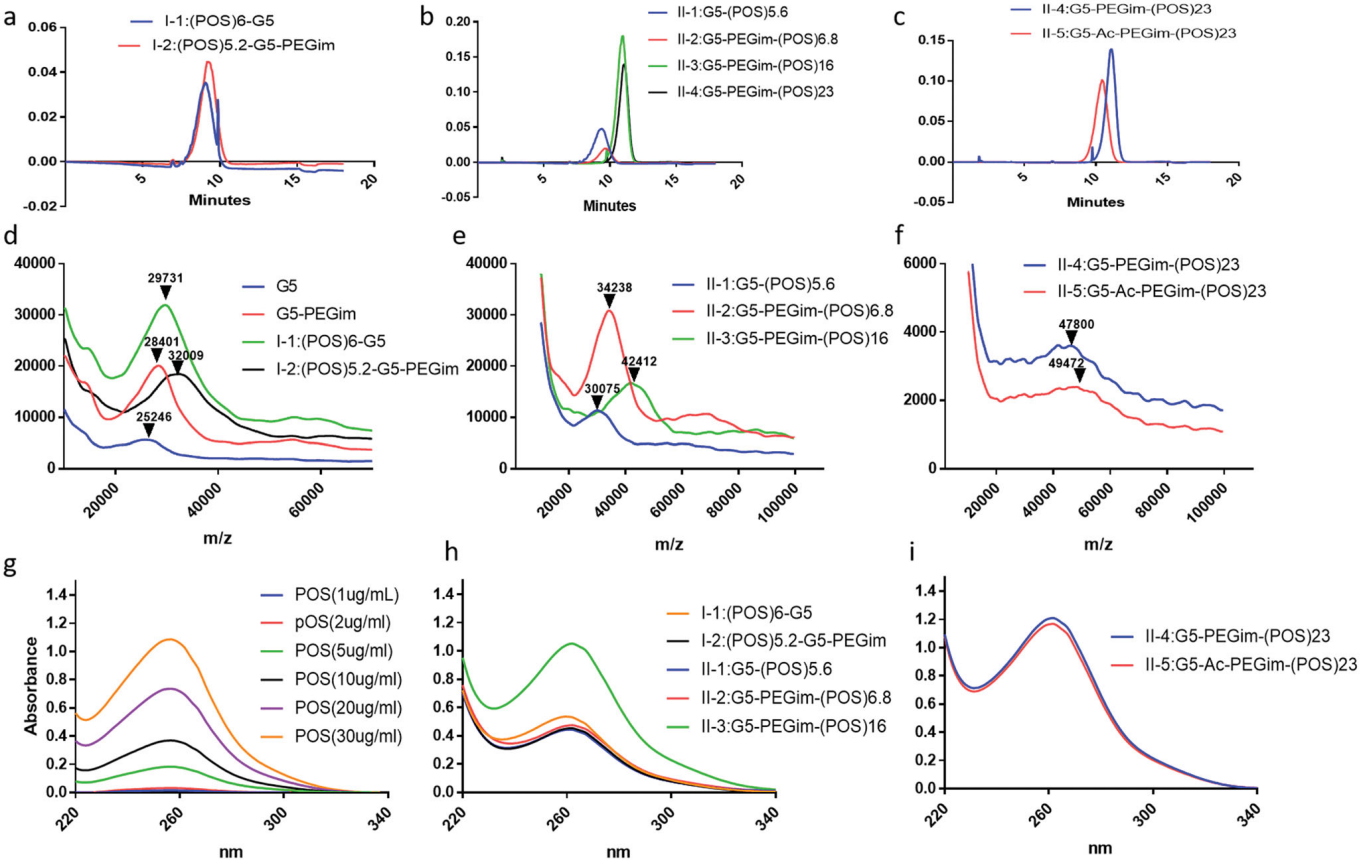
(a–c) HPLC chromatogram of series of G5 POS conjugates under UV 265 nm. (d–f) MALDI-TOF mass spectra of G5, imidazole-modified G5 (G5-PEGim), and various G5 POS conjugate **I-1**, **I-2**, **II-1**, **II-2**, **II-3**, **II-4**, and **II-5**. (g–i) UV–vis spectra of POS at various concentrations and series of G5 POS conjugates at 0.1 mg/mL.

HPLC has been used as a vital tool to analyze PAMAM dendrimers and their conjugates (Islam et al., 2005; Shi et al., 2006). In this study, we used HPLC to analyze the synthetic POS derivatives and evaluate the purity and homogeneity of these series of conjugates. Since G5 dendrimers have no UV absorption, after conjugation with POS, which has a maximal UV absorption at approximately 260 nm, the synthetic series of G5 POS conjugates were analyzed by HPLC with detection at 265 nm (Figure 3(a–c)). The HPLC analysis suggested that these POS derivatives showed distinguishable retention times (RT) (Supporting Information-3), and the purity of the series of class **I** G5 POS conjugates was higher than 98%. With the same purification procedure, performed by precipitation in acetonitrile and then dialyzed using a membrane tubing (MWCO = 10 kDa), class **II** conjugates (POS derivative **D**) were even purer than class **I** conjugates.

### 3.6. MALDI-TOF mass spectrometry

As an important technique for characterization of dendrimers and their conjugates, MALDI-TOF mass spectrometry detects the average molecular weight, providing the information about the success of each conjugation reaction and the number of attachments. Figure 3(d–f) shows the MALDI-TOF mass spectra of the various G5 POS conjugates. The molecular weights increased through each covalent process due to additional molecular attachments. The average number of POS attached to the dendrimer can be calculated by dividing increasing molecular weights after attachment by the molecular mass of the corresponding POS (for **I-1**, **I-2**) or POS derivative **D** (for **II-1**, **II-2**, **II-3**, **II-4**) **(**Figure 1(c)).

To class **I** conjugates, POS was reacted with G5 dendrimer or G5-PEGim to make conjugate **I-1** or **I-2**. With the molar ratio of POS/G5 = 10, the mean numbers of POS attached to the dendrimer or G5-PEGim were 6 or 5.2, respectively. To class **II** conjugates, POS derivative **D** was reacted with G5 dendrimer or G5-PEGim with varying molar ratios of POS/G5 (10, 20, and 30) to produce various numbers of attachment. After calculation, the mean number of POS attachments for **II-1** to **II-4** was 5.6, 6.8, 16, and 23, respectively. The final conjugate **II-5** was obtained by neutralization of any unreacted primary amines with acetic anhydride. Based on the increasing molecular weights from **II-4** to **II-5**, there were 40 acetyl groups attached to the dendrimer surface. As expected, the acetylation reaction was incomplete due to steric hindrance of the surface amines, which prevents some of the residues from reacting (Maiti et al., 2004).

### 3.7. Ultraviolet–visible spectroscopy

Using the absorption peak of POS at 260 nm in synthetic conjugates, UV–vis spectroscopy (Figure 3(g–i)) demonstrated the ratio of the conjugation of POS to G5 dendrimer in the conjugates. POS and its derivatives were analyzed in a 50% methanol aqueous solvent; however, given the high solubility, the synthetic G5 POS conjugates were dissolved in 10-fold-concentrated PBS (pH 7.4) for evaluation. Using UV–vis absorption spectra, the average number of attached POS molecules was determined by comparing the maximal absorption of these conjugates with a standard POS solution using a POS calibration curve of absorbance vs. concentration at 260 nm (Supporting Information-4). The numbers were consistent with what was detected by MALDI-TOF spectrometry (Figure 1(c)).

### 3.8. Release kinetics of targeting conjugate II-5 in human plasma

**Figure 4.**
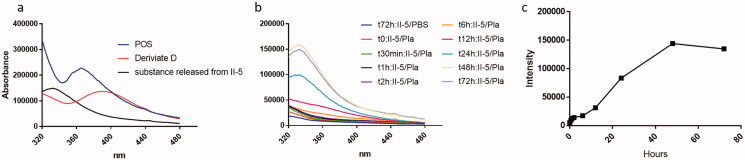
(a) Representative of the emission spectrum (excited at 260 nm) of POS, POS derivative **D** and the released substance from conjugative **II-5**, (b) emission spectrum for outer media of G5 POS conjugate **II-5** in PBS and plasma (Pla) in membrane dialysis release studies. (c) *In vitro* release profile of G5 POS conjugate **II-5** in human plasma.

The *in vitro* POS release from G5 dendrimer via hydrolysis was investigated in human plasma. The conjugate **II-5** (2.4 mg) was dissolved in 0.5 mL of PBS (pH = 7.4) mixed with 0.5 mL of human plasma, then placed in a dialysis bag, with an MWCO of 10,000, and immersed in 19 mL of PBS. The samples were placed in a bath tank at 37 °C while stirring. The outer media was sampled at predetermined time points by removing 1.5 mL and an equal volume of fresh PBS was replenished. With the 10 kDa membrane, only the small molecule POS released from conjugate **II-5** was able to diffuse across the membrane and into the outer buffer, which was then measured by a fluorimeter. As demonstrated in Figure 4(a), due to the intermolecular bonds altering the spectral characteristics after excitation at 260 nm, POS showed fluorescent spectra at 365 nm, while its derivative **D** had a peak at 395 nm and the modified material released from conjugate **II-5** peaked at 333 nm. Compared in PBS, conjugate **II-5** in plasma released a substance relative to POS continuously over a 72-hour period (Figure 4(b)). The fluorescence intensity at 333 nm approached its maximal value within several hours and remained relatively stable over a period of 72 h. The readings suggested that about half of POS was released from the conjugate in 24 h and the concentration of released POS reached the highest level at 48 h (Figure 4(c)).

### 3.9. *In vitro* antifungal activity

**Figure 5.**
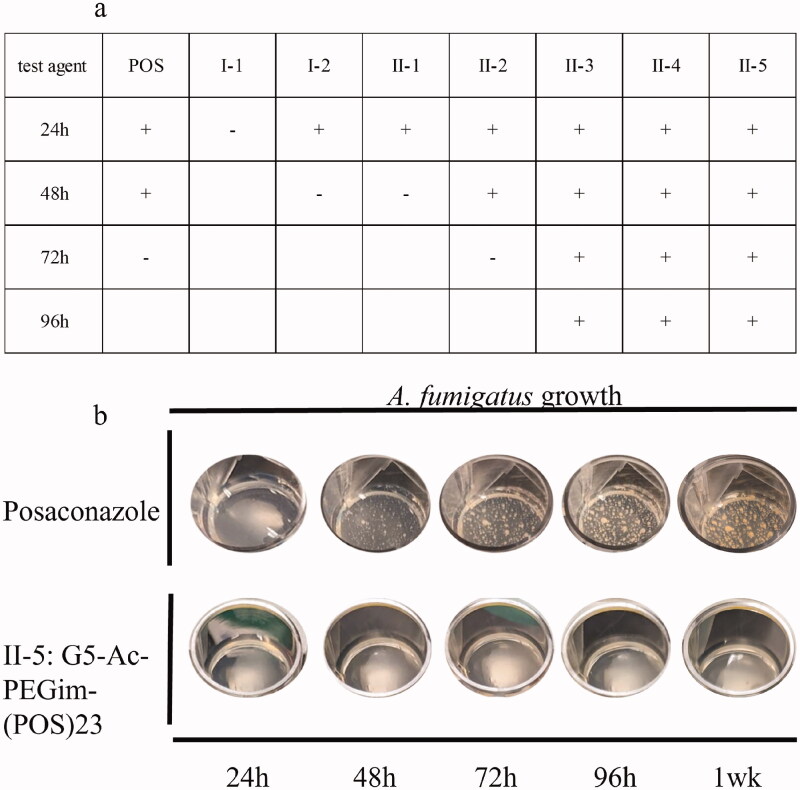
(a) Concentration of POS is 25 µg/mL and concentration of each G5 POS conjugate is equivalent to 25 µg/mL of free POS. (+) indicates the inhibition with the growth of *A. fumigatus* in the timepoint; (–) indicates no inhibition with the growth of *A. fumigatus* in the timepoint. (b) Antifungal index against *A. fumigatus*: posaconazole v G5 POS conjugate **II-5**.

All G5 POS conjugates, class **I** (**I-1** to **I-2**) and class **II** (**II-1** to **II-5**), were tested on *A. fumigatus* to determine their antifungal activity as compared to free POS (Figure 5(a)). The results indicate that: (i) conjugate **I-1** and **I-2**, in which POS molecules were directly coupled to G5 dendrimer, were less effective than conjugates **II-1** to **II-5**, in which the POS derivative **D** molecules were coupled to G5 dendrimer through spacers; (ii) in comparison with non-modified G5 conjugation (**I-1** and **II-1)**, G5-PEGim conjugates (**I-2**, **II-2**) showed more effective antifungal activity; (iii) conjugates **II-3** and **II-4** exhibited even more efficient inhibition of *A. fumigatus* growth over 96 h than conjugate **II-2** due to more POS attachments. Further observations indicated that the conjugate **II-5** was able to inhibit the growth of fungi through an entire a week (Figure 5(b)).

### 3.10. Stability in PBS solution

POS and its derivative **A**, **B**, **C**, and **D** were dissolved in small volumes of methanol and then diluted with 10-fold-concentrated PBS (pH 7.4) for a 0.1 mg/mL solution. G5 POS conjugate **I-1**, **I-2**, **II-1**, **II-2**, **II-3**, **II-4**, and **II-5** were dissolved in PBS for a final concentration of 0.5 mg/mL. The resulting solutions were incubated at 33 °C for a total of 1 week. A sample was taken every 24 hours for HPLC analysis (Supporting Information-5). The HPLC analysis showed that the synthetic POS derivatives and G5 POS conjugates demonstrated incredible stability. After one week incubation at 33 °C, the HPLC spectrums for these synthesized compounds did not show any major peak changes. However, a reduction in peak area was seen over time, particularly in derivative **C** from time 0 to 24 h; these reductions might be due to the evaporation of methanol from the PBS buffer solution decreasing the solubility of POS and its derivatives which causes a loss in concentration. Loss of peak area does not occur in conjugates **I-1**, **I-2**, **II-1**, **II-2**, **II-3**, **II-4**, and **II-5** as the PBS solutions because these conjugates are highly soluble in PBS. The results demonstrated that both the synthetic POS derivatives and conjugates were stable in PBS.

## 4. Conclusions

Several of the POS derivatives and a series of G5 dendrimer POS conjugates have been synthesized and analyzed. POS has been coupled to dendrimer either directly or through a short linker by carbamate bond. Further, conjugates were prepared with two types of G5 dendrimer: standard amine terminated G5 and PEG-imidazole modified G5. Our antifungal studies have shown that conjugates using PEG-imidazole modified G5 as a platform were most effective when compared with conjugates with non-modified G5. Furthermore, indirect conjugates, where POS was coupled through diglycolamine spacers, show greater antifungal activity than direct conjugates. With 23 molecules of POS coupled to G5-PEGim, conjugate **II-5** shows antifungal activity over a period of 96 hours to 1 week, which corresponds to its release behavior in human plasma.
